# Sexual attraction enhances glutamate transmission in mammalian anterior cingulate cortex

**DOI:** 10.1186/1756-6606-2-9

**Published:** 2009-05-06

**Authors:** Long-Jun Wu, Susan S Kim, Xiangyao Li, Fuxing Zhang, Min Zhuo

**Affiliations:** 1 Department of Physiology, University of Toronto, 1 King's College Circle, Toronto, Ontario M5S 1A8, Canada

## Abstract

Functional human brain imaging studies have indicated the essential role of cortical regions, such as the anterior cingulate cortex (ACC), in romantic love and sex. However, the neurobiological basis of how the ACC neurons are activated and engaged in sexual attraction remains unknown. Using transgenic mice in which the expression of green fluorescent protein (GFP) is controlled by the promoter of the activity-dependent gene *c-fos*, we found that ACC pyramidal neurons are activated by sexual attraction. The presynaptic glutamate release to the activated neurons is increased and pharmacological inhibition of neuronal activities in the ACC reduced the interest of male mice to female mice. Our results present direct evidence of the critical role of the ACC in sexual attraction, and long-term increases in glutamate mediated excitatory transmission may contribute to sexual attraction between male and female mice.

## Introduction

Romantic love is a near universal notion, and is associated with a specific set of physiological, psychological and behavioral traits, most of which are also characteristic of mammalian courtship attraction [1]. The olfactory system has been the focus of the majority of mammalian studies, as it is essential for sex-specific behaviors such as sex discrimination, sexual attraction, mating and aggression [2-6]. In humans, numerous studies have been conducted in order to determine the neural correlates associated with romantic love. Using imaging techniques such as positron emission tomography (PET) or functional magnetic resonance imaging (fMRI), higher brain structures such as primary and associative cortical regions, including piriform, orbitofrontal, temporal and cingulate cortices have been linked to sexual arousal and romantic love [7,8]. Therefore, sexual behaviors are thought to be associated with neural networks of cortical activation.

The anterior cingulate cortex (ACC) is one of the major cortical areas involved in both negative (such as pain and fear memory) and positive (such as pleasure and sexual arousal) affective states [9-12]. For positive affective states, activation of the ACC has been correlated with romantic love, sexual arousal, as well as sexual drive [13-16]. An earlier study also reported that electrical stimulation of sites within the ACC of macaque induced penile erections [17], suggesting the direct role of the ACC in sexual arousal. With the use of immediate early genes like *c-fos *as a marker, neurons in the ACC have been shown to be activated after sexual stimulation in rodents [18,19]. However, the neurobiological basis of how the ACC neurons are activated and engaged in sexual attraction remains poorly understood. One potential reason is that it is difficult to identify ACC neurons that are activated by sex or sexual attraction in electrophysiological experiments. In the present study, we performed whole-cell patch- clamp recording from neurons in brain slices of transgenic mice in which the expression of green fluorescent protein (GFP) is controlled by the promoter of the activity-dependent gene *c-fos*. Thus, we were able to record in the first time from ACC pyramidal cells that are responsive to female mice. We found that the sexual attraction between male and female mice indeed triggered long-lasting enhancement of glutamate mediated excitatory transmission within the ACC synapses.

## Results

### Two chamber behavioral test for male-female attraction

**Figure 1:**
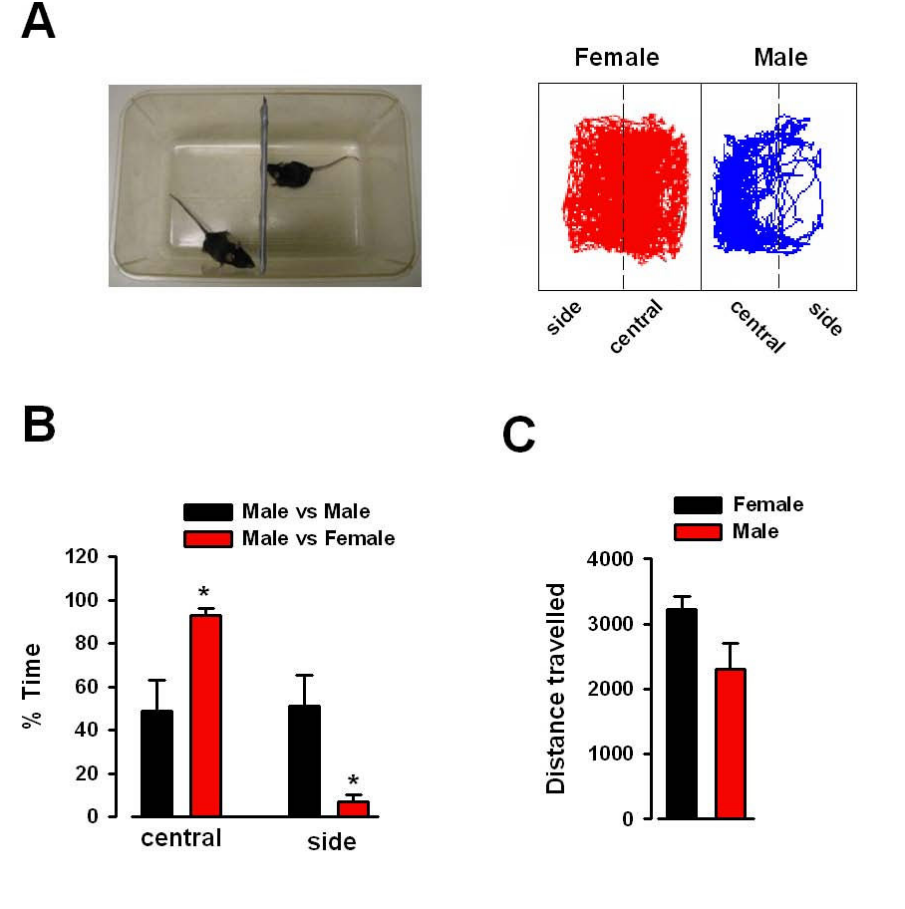
**Behavioral test for sexual attraction**. (A) Photograph and diagram of the sexual attraction test. The two boxes are further divided into side and central zones as indicated by the dashed line. Representative traces showing the movement of the male and female mice for 30 min. Left = female (red); right = male (blue). (B) Male mice (n = 5) spent significantly more time in the central zone than in the side zone (male vs. female) (n = 5), while there is no difference in the time spent in either zone in male vs. male test. * P = 0.007. (C) There is no difference in the total distance traveled between male and female mice in the sexual attraction test.

It has been reported that pheromones are the major sexual signals used by male mice [20-22]. To investigate the sexual attraction between male and female mice, we designed a behavioral test to evaluate the interactions between the animals. The testing cage was divided in two by a porous divider, with each side further divided into central and side areas. The mice were allowed to freely roam for 30 min and were monitored by a tracking camera (Fig. 1A). We found that the male mice (n = 5) spent significantly more time in the central area than in the side further away from the female mice (n = 5) (P = 0.007, Fig. 1B). However, when two non cage mate male mice were tested, we found no such differences for they spent time in the two areas equally, suggesting that while male mice are sexually attracted to female mice, no such attraction exists between the two male mice (Fig. 1B). To determine whether the differences in time spent in central and side areas were due to hyperactivity, the total distance traveled was examined. We found no differences between male and female mice (Fig. 1C), indicating that the behavioral responses of male and female mice to the testing apparatus were indistinguishable.

### The ACC is activated and required for sexual attraction

**Figure 2:**
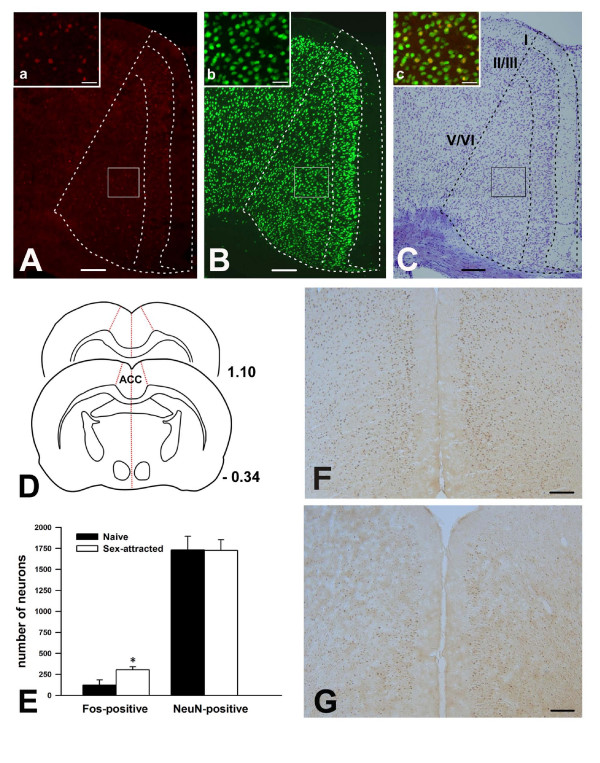
**Sexual attraction activated Fos expression in the ACC neurons in male mice**. (A) Fos immunostaining in the ACC after sexual attraction. The framed area is magnified in (a). (B) NeuN immunostaining in the ACC. The framed area is magnified in (b). (C) The same section as shown by (A, B), was counterstained by Nissl technique; demarcation for neuronal layers in the ACC. The inset (c) is the overlapping of (a) and (b). Yellow color in (c) represents the Fos/NeuN double-labeled neurons. (D) Diagram showing the ACC from Bregma -0.34 to 1.1, where the Fos-positive and NeuN positive cells were counted. (E) Bar graph showing the significant increase of Fos neurons in layer II/III and ACC after sexual attraction test compared to naïve mice. (F and G) The representative slices with Fos staining between sex-attracted group (F) and naïve mice (G). The increased Fos expression was found in layer II/III and layer V/VI. Bar = 150 μm in A – C, F and G; and 35 μm in a – c.

The ACC is well known to be involved in sexual arousal [10,13,14]. However, its molecular substrates still remain unknown. To determine the role of the ACC in sexual attraction in mice, we first used immunohistochemistry to identify ACC activation following sexual attraction. It has been shown that Fos could be used as a marker for neuronal activation following sexual stimulations [19,23]. To quantify neuronal activation in the ACC by sexual attraction, we used double immunostaining of Fos and NeuN (Fig. 2A and 2B). We counted activated neurons (Fos-positive) and total neurons (NeuN-positive) in different layers of the ACC, which was determined by Nissl staining (Fig. 2C). We found that the number of activated neurons was significantly enhanced in layers II/III and V/VI of male mice after exposure to female mice compared to male mice that had been exposed to non-cage mate male mice (Table 1, Fig. 2E–G). However, there was no difference in the number of activated neurons in layer I of the ACC (Table 1). These results indicate that sexual attraction causes selective activation of the neurons in the ACC including pyramidal cells.

**Table 1:** Density of Fos-immunostained (fos +), NeuN-immunostained (NeuN+) neurons and percentages of double-labeled neurons in the ACC.

		**Number of immunostained cells**		**Double labeling**	
			
		**Fos +**	**NeuN +**	**(% of NeuN +)**	**(% of Fos +)**
I	Naïve	2.8 ± 2.0	32.0 ± 5.4	8.1 ± 5.4	100
	Sex-attracted	3.8 ± 1.7	34.8 ± 4.6	11.2 ± 5.2	100
II/III	Naïve	52.0 ± 26.9	642.0 ± 64.6	8.1 ± 4.2	100
	Sex-attracted	135.2 ± 13.1 (**)	678.2 ± 66.9	21.4 ± 3.9 (**)	100
V/VI	Naïve	67.0 ± 34.2	1057.4 ± 99.1	6.1 ± 3.0	100
	Sex-attracted	166.8 ± 23.0 (**)	1011.8 ± 65.5	17.2 ± 3.2 (**)	100

Values represent mean ± SEM, comparable sections of 25 mm thick between two groups of mice were used for quantification (3 sections per mouse, n = 5 for each group). Asterisks indicate significant difference between control and sex-attracted groups (P < 0.05, n = 5).

**Figure 3:**
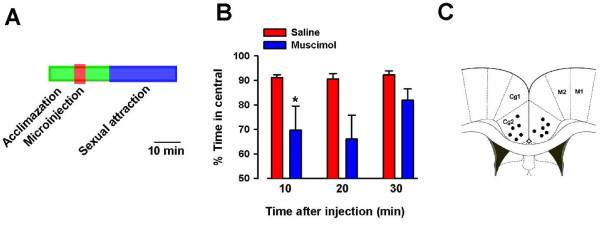
**The ACC is required for sexual attraction**. (A) Experimental design for the behavioral experiment after musiimol injection. (B) Local injection of muscimol in the ACC significantly reduced sexual attraction in male mice 10 minutes after injection. The sexual attraction was recovered within 30 minutes. (C) Location of injection sites from all animals included in the study.

We then performed pharmacological manipulations to see if the activation of the ACC is necessary to affect sexual attraction between male and female mice. It is known that local injection of muscimol, a selective agonist for GABA_A _receptors, could inactivate the ACC neuronal activity [24]. We found that bilateral injections of muscimol (0.5 μl per side) into the ACC of male mice significantly reduced sexual attraction in the first 10 min (Fig. 3A and 3B). The inhibitory effect is time-dependent, with the effect of muscimol almost completely diminished at 30 minutes after the injection (Fig. 3B).

### Altered synaptic transmission in the ACC neurons after sexual attraction

**Figure 4:**
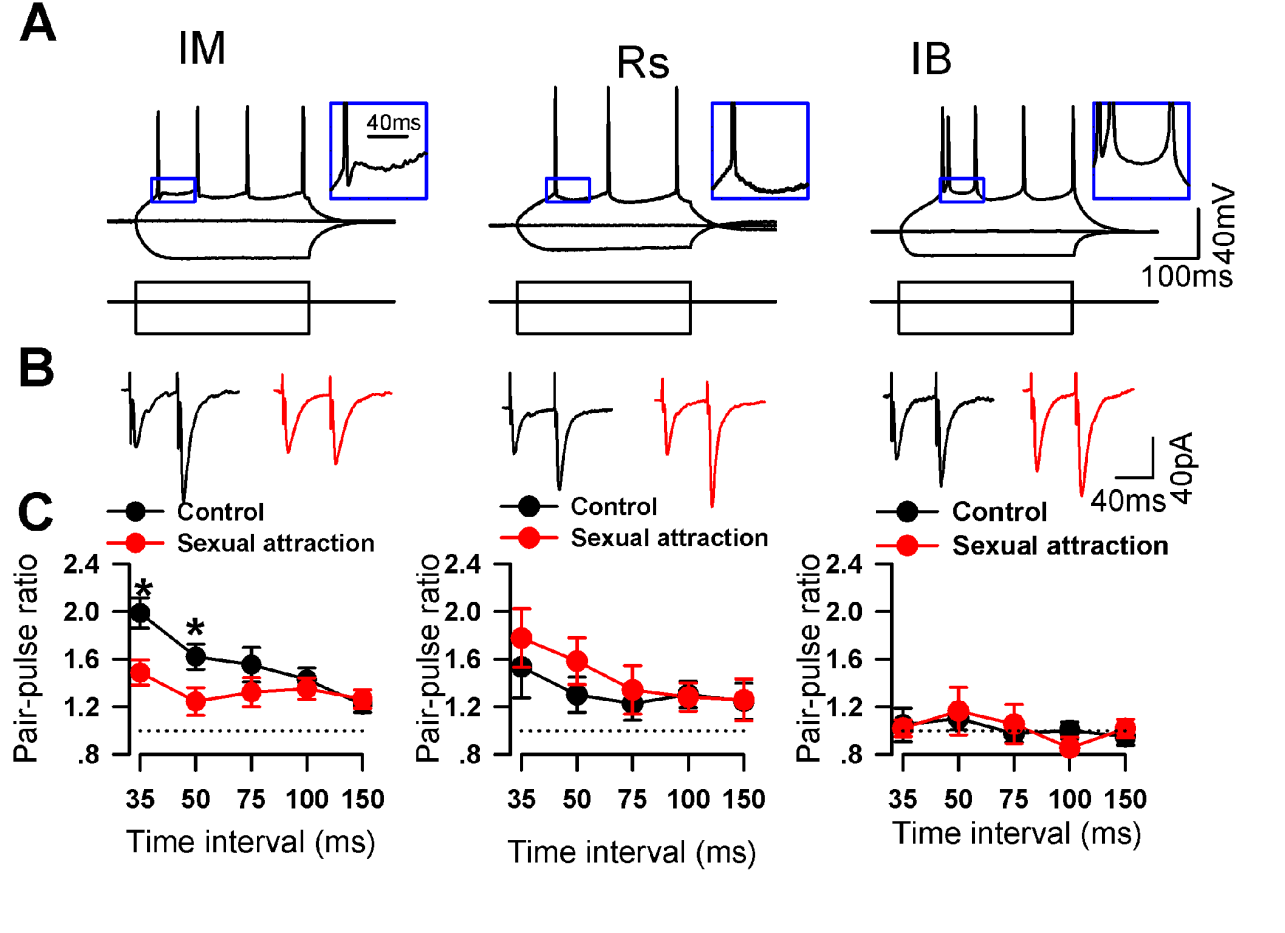
**PPF was changed in the ACC after sexual attraction**. (A) Sample traces show the three types of pyramidal neurons in layer II/III of ACC. IM, intermediate neurons; RS, regular spiking neurons; IB, intrinsic bursting neurons. (B) PPF of IM neurons (upper) was decreased after sexual attraction in the pathway of Layer V-Layer II/III, while no change has been detected on other two kinds of pyramidal neurons. (C) No interaction between two pathways induced by layer V and layer I stimulations. Time interval between two stimulations was 55 ms. (D) PPF induced by S1 (layer I stimulation) did not change after sexual attraction.

**Figure 5:**
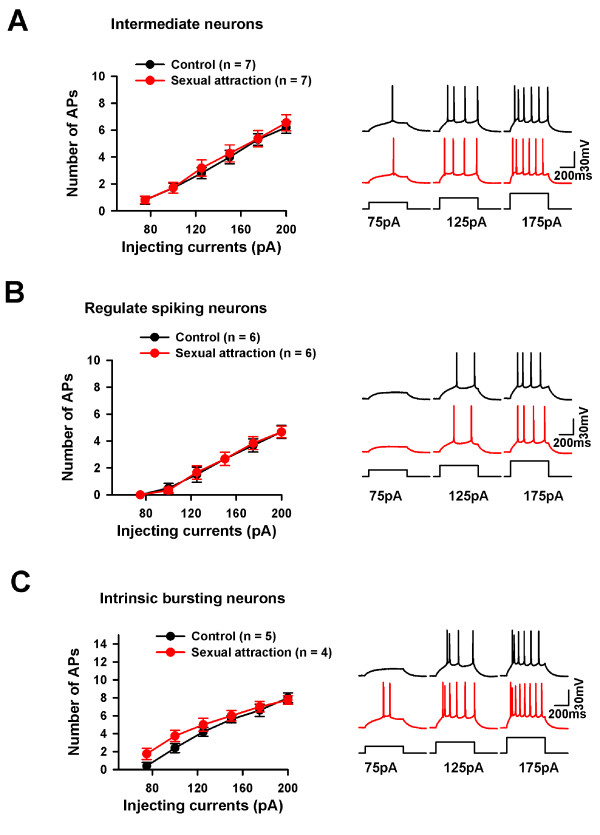
**The excitability of pyramidal neurons was not changed after sexual attraction**. (A) Action potentials were induced by current injection from 50 pA to 200 pA with 25 pA step. The number of spikes was counted at different current injections to measure the excitability of neurons. There was no change in the excitability of IM neurons between two groups. (B) No change in the number action potentials in RS neurons between two groups. (C) No change in the number action potentials in IB neurons between two groups.

To study the cellular mechanisms of neuronal activation in the ACC after sexual attraction, we then performed whole-cell patch-clamp recordings in the ACC pyramidal neurons. Based on the firing properties, pyramidal neurons in the ACC were classified into three categories: (i) regular spiking (RS) cells, in which the single spike is followed by a slow afterhyperpolarization (AHP), intermediate (IM) cells, in which the single spike is followed by a fast AHP and a fast afterdepolariztion (ADP), and intrinsic bursting (IB) cells that fired an initial spike doublet (Fig. 4A). We first examined if neuronal firing properties were altered after sexual attraction. We compared the number of firing action potentials after current injection in naïve mice and mice that had undergone the sexual attraction test. Three types of pyramidal neurons were recorded in both group and we found no difference in any of them (Fig. 5). These results indicate that there are not obvious significant changes in neuronal firing properties in ACC pyramidal cells, although we cannot completely rule out other changes that may not be detected under the current testing protocol.

We then investigated excitatory synaptic transmission by recording the excitatory postsynaptic currents (EPSCs) in layer II/III pyramidal neurons by focal stimulation in deeper layer V in the presence of GABA_A _antagonist, picrotoxin (100 μM). Paired-pulse facilitation (PPF) ratio, a commonly used criteria for studying presynaptic release [25,26], was examined in three types of ACC pyramidal neurons. We found that in RS cells and IB cells, there was no difference in PPF between naïve mice and mice after sexual attraction (IB: F_1:44 _= 0.008, P = 0.927; RS: F_1:49 _= 1.206, P = 0.279, Two-way ANOVA, Fig. 4B and 4C). However, a significant decrease in PPF was found in IM cells of sexually attracted mice compared to that of naïve mice (F_1:89 _= 9.825, P = 0.002, Fig. 4B and 4C). These results indicate that synaptic transmission was altered in the ACC neurons after sexual attraction, especially IM cells.

### The alteration in PPF is input-specific

**Figure 6:**
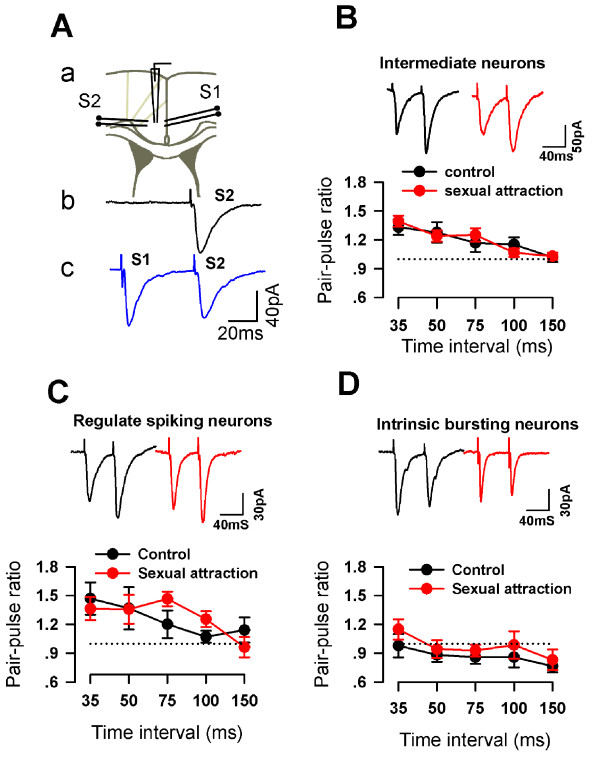
**PPF in the pathway of Layer I-Layer II/III were not changed after sexual attraction**. (A) No interaction between two pathways induced by layer V and layer I stimulations. Time interval between two stimulations was 55 ms. (B, C and D) PPF induced by S1 (layer I stimulation) did not change after sexual attraction.

To determine whether the change in PPF was input-specific, we performed two-pathway experiments, recording the EPSCs in layer II/III IM cells by stimulating in deep layer V (S2) and layer I (S1) of ACC slices (Fig. 6Aa). First, we tested the independence of the two pathways. We compared S2-induced EPSCs in the absence or presence of preceding S1 stimulation. There was no difference in the amplitude of S2-induced EPSCs with or without S1 (the ratio is 1.01 ± 0.07, n = 4, Fig. 6Ab, c). Also there was no difference in the amplitude of S1-induced EPSCs with or without S2 (n = 4). These results suggest that there is no overlap between the two stimulation pathways. Then we tested PPF of S1-induced EPSCs and S2-induced EPSCs in sexually attracted and naïve mice. We found that while S2-PPF was decreased, no such change was seen in S1-PPF between the two groups in IM cells (Fig. 6B). Moreover, there was no difference in S1-PPF between the two groups in either RS or IB cells (Fig. 6C and 6D). Therefore, the alteration of synaptic transmission to IM cells was input-specific and likely mainly occurred in synaptic connections from presynaptic fibers located in layer V or passing through layer V to layer II/III.

### Enhanced synaptic transmission to activated neurons in the ACC after sexual attraction

**Figure 7:**
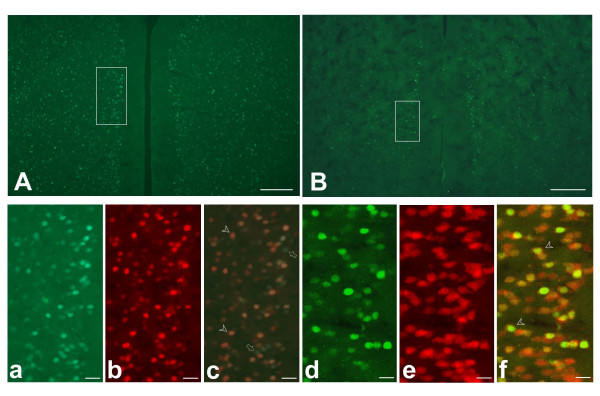
**GFP is co-stained with Fos protein and NeuN in the ACC of transgenic mice**. (A) The GFP-positive cells in the ACC after sexual attraction are overlapped with Fos-positive cells. The framed area was used for magnification in a-c. (B) Some of GFP-positive cells in the ACC after sexual attraction are overlapped with NeuN-positive cells. The framed area was used for magnification in d-f. Bar = 200 μm in (A, B), 30 μm in (a – c) and 20 μm in (d – f).

Considering the heterogeneous cells in the ACC, it is important to identify cells that are responsive to the sexual attraction. Next, we took advantage of the transgenic mice in which the expression of GFP is controlled by the promoter of *c-fos *gene [27]. We found that GFP-positive neurons were widely observed in the ACC of male mice exposed to female mice. To confirm that these positive neurons do express the Fos protein, we performed immunostaining with the antibody. We found that GFP-positive cells were also Fos-positive (Fig. 7A). These GFP-positive neurons were also co-stained with the antibody for NeuN, a typical neuronal marker (Fig. 7B). Therefore, the transgenic mice were appropriate for studying neuronal activation in the ACC following sexual attraction.

**Figure 8:**
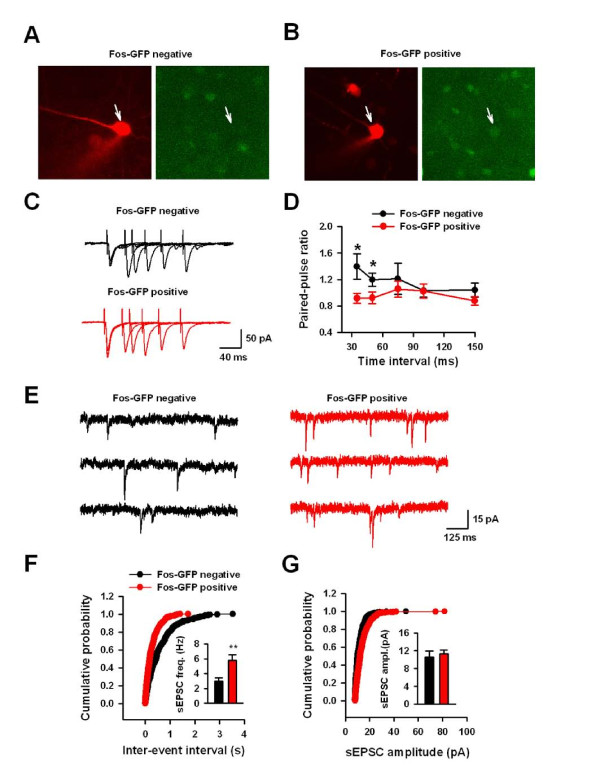
**Increased presynaptic glutamate release to Fos-GFP positive neurons after sexual attraction**. (A and B) Example of Fos-GFP negative (A) and positive (B) cells from layer II/III of the ACC that was targeted for whole-cell recording dilated with Alexa fluor 594. (C) Typical traces showing paired-pulse stimulations with interval of 35, 50, 75,100, 150 ms in Fos-GFP negative and positive neurons. (D) Decreased paired-pulse facilitation in Fos-GFP positive neurons at interval 35 ms or 50 ms compared with those in Fos-GFP negative neurons. (E) Representative traces showing mEPSC recordings in Fos-GFP negative and positive neurons. (F) Cumulative probability and pooled data (inset) showing the increased mEPSC frequency in Fos-GFP positive neurons than that in Fos-GFP negative neurons. (G) Cumulative probability and pooled data (inset) showing that there is no difference in mEPSC amplitude between Fos-GFP positive and negative neurons.

**Figure 9:**
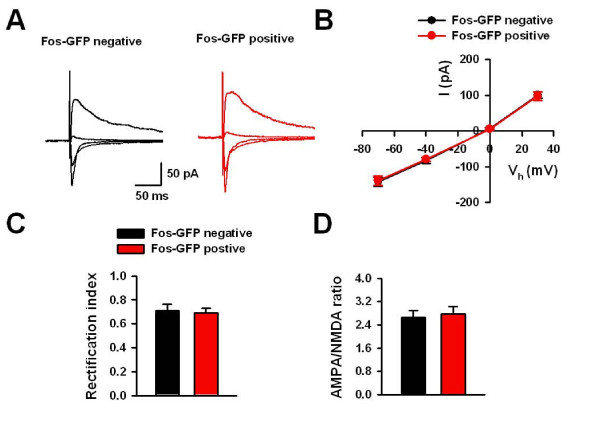
**The postsynaptic responses in Fos-GFP positive neurons after sexual attraction**. (A) Typical traces of EPSCs at holding potentials of -70, -40, 0 and 30 mV in Fos-GFP negative and positive neurons. (B) The pooled data showing the normal I–V responses in Fos-GFP positive neurons. (C and D) The rectification index (C) and AMPA/NMDA ratio (D) for EPSCs was similar in Fos-GFP positive and negative neurons.

To study the molecular mechanism of neuronal activation in the ACC, we examined the glutamatergic transmission in GFP-positive neurons by using fosGFP mice. EPSCs were recorded in fos-positive or fos-negative neurons in layer II/III by evoking in layer V (Fig. 8A and 8B). At different holding potentials, we measured the peak current which is mainly mediated by AMPA receptors, and the current 50 ms after the peak, which is mainly mediated by NMDA receptors. We found no differences in the current-voltage (I–V) relationship of AMPA EPSCs between GFP-positive and negative neurons (n = 8 neurons/4 mice from each group, P > 0.05) (Fig. 9A and 9B). In addition, there was no difference in rectification index of AMPA current (0.69 ± 0.04 vs. 0.71 ± 0.05; n = 8 neurons/4 mice from each group, P = 0.73) or AMPA/NMDA ratio (2.8 ± 0.3 vs. 2.7 ± 0.2; n = 8 neurons/4 mice from each group, P = 0.75) (Fig. 9C and 9D). These results suggest that the function of postsynaptic AMPA receptors is unlikely to be altered.

We then compared PPF of GFP-positive and GFP-negative neurons (Fig. 8C and 8D). Consistently, we found significant decreases of PPF at 35 ms and 50 ms intervals in GFP-positive neurons (35 ms interval, 0.92 ± 0.08 vs. 1.40 ± 0.19; n = 8 neurons/4 mice in each group, P < 0.05, Fig. 8E and 8F). Although we did not confirm if recorded GFP-positive neurons are IM cells, our recent data found that about 50% layer II/III pyramidal cells are IM cells (unpublished data). To confirm the possibility of enhanced presynaptic glutamate release in GFP-positive neurons, we examined the miniature EPSCs (mEPSCs). We found that mEPSC frequency in GFP-positive neurons (5.8 ± 0.8 Hz, n = 8 neurons/4 mice) was larger than in GFP-negative neurons (3.0 ± 0.5 Hz, n = 8 neurons/4 mice) (P < 0.01, Fig. 8E and 8F). However, there was no significant difference in the amplitude of mEPSCs between GFP-positive and negative neurons (n = 8 neurons/4 mice, P = 0.64, Fig. 8E and 8G). These results indicate that presynaptic glutamate release was increased in the GFP-positive neurons.

## Discussion

In the present study, we focused on sexual attraction and the ACC, a region known to be associated in pain and pleasure [11]. We found that the ACC is robustly activated in male mice after exposure to female mice, as demonstrated by Fos expression, and pharmacological inhibition of the ACC could reduce sexual attraction. Although the excitability of ACC pyramidal neurons was not altered, the excitatory synaptic transmission was enhanced after sexual attraction. By using transgenic fosGFP mice, we found that sexual attraction induced functional increases of presynaptic glutamate release to activated neurons in ACC slices. Our results demonstrate the critical role of the ACC in sexual attraction, and provide the first evidence for the cellular mechanism of the ACC neurons involved in the sexual attraction.

Evidence from animal studies indicates that the central supraspinal systems controlling sexual arousal are localized predominantly in the limbic system. The ACC has been implicated as an unique area for evoking affective state such as romantic love [15], and a PET imaging study showed that the ACC is specifically activated during the excitation phase of sexual stimuli in men [7]. In the present study, we used Fos expression as a marker of functional activation of neurons in the ACC. We found that all layers except layer I are activated bilaterally in the ACC, suggesting that sexual attraction results in specific pattern of activation. The results are consistent with previous reports mapping the brain areas involved in sexual attraction [19,28], and establish the critical roles of the ACC in the sexual behaviors. However, due to limitations in using Fos as a marker, future studies must be conducted to elucidate the exact inputs to and outputs from the ACC after activation by sexual attraction. The significance of the activation of the ACC is probably not on the perception of sexual stimulation, which is fulfilled by rodent olfactory systems. Rather, we propose that the activation of the ACC affects positive emotional state, leading to sexual behaviors. In favor of the role of the ACC in sexual attraction, we found that inactivation of the ACC by local infusion of muscimol reduced the interest of male mice to female mice.

How could the ACC neurons be activated by sexual attraction? Fos expression is due to the conversion of extracellular signals into early genomic activation, which indicates that a signal transduction event has taken place in those activated neurons. Therefore, we hypothesized that functional alterations occurred in the activated neurons in the ACC after sexual attraction. To test this, we used fosGFP mice, a useful tool able to link sensory stimulation in animals to functional neuroanatomy and electrophysiology. In brain slices, we could detect GFP signals and performed whole-cell patch clamp recordings in GFP-positive neurons. We found enhanced presynaptic glutamate release, as shown by decreased PPF and increased mEPSC frequency. However, postsynaptic response was unlikely to be altered, since no changes were observed in the response mediated by either AMPA or NMDA receptors. We propose that the increased presynaptic glutamate release is the main trigger for the activation of neurons in the ACC following sexual attraction. This raises two essential questions which will require further insight into the function of the ACC in sexual attraction: (1) what signal triggers the increased glutamate release? and (2), what are the functional consequences of Fos activation?

In sum, our results provide a first mouse electrophysiological model for studying the sexual attraction in forebrain areas. At a single cell level, we are able to demonstrate a long-lasting changes in glutamate mediated excitatory synaptic transmission within the ACC, a cortical region known to be critical for sexual desire, attraction, and romantic love in human imaging studies. The use of transgenic and gene knockout mice in future will provide powerful tools to explore cellular and molecular basis of key brain functions of human such as love, sex and attraction between man and woman.

## Methods

### Animals

Adult C57BL/6 mice were purchased from Charles River (9–17 weeks old). The transgenic fosGFP mice were obtained from laboratory of Dr. Alison Barth (Carnegie Mellon University). All mice were maintained on a 12 h light/dark cycle with food and water provided *ad libitum*. All protocols used were approved by The Animal Care and Use Committee at the University of Toronto.

### Sexual Attraction Test

We devised a novel test to measure the degree of sexual attraction. The testing apparatus consisted of a rectangular box with a divider in the middle. The divider had numerous holes that were large enough to allow for vision and olfaction yet small enough to prevent physical interaction. For each test, mice were placed on opposing sides of the divider and were allowed to move freely within the box for 30 minutes. The more time the animal spent in the central area (area closer to the opposite sex), the greater the sexual attraction. The movement of the mice were tracked and recorded by a video camera tracking system (Ethovision, Noldus VA). Each mouse was individually placed in the rectangular box prior to testing for 10 minutes for acclimatization. None of the animals, whether male or female, were sexually experienced prior to the experiment. Although we did not monitor the female hormone cycle stage, we found similar sex attractions between male and female mice.

### Whole-cell patch-clamp recordings

Coronal brain slices (300 μm) containing the ACC from six- to eight-week-old male mice were prepared using standard methods [29]. Slices were transferred to a submerged recovery chamber with oxygenated (95% O_2 _and 5% CO_2_) artificial cerebrospinal fluid (ACSF) containing (in mM): 124 NaCl, 2.5 KCl, 2 CaCl_2_, 2 MgSO_4_, 25 NaHCO_3_, 1 NaH_2_PO_4_, 10 glucose at room temperature for at least 1 hour.

All electrophysiological experiments were performed at room temperature. An Olympus BX51WI microscope (Tokyo, Japan) with infrared DIC optics was used for visualization of whole-cell patch clamp recording. Excitatory postsynaptic currents (EPSCs) were recorded from layer II/III neurons with an Axon 200 B amplifier (Molecular devices, CA) and the stimulations were delivered by a bipolar tungsten stimulating electrode placed in layer V of the prefrontal slices. EPSCs were induced by repetitive stimulations (duration is 200 μs, intensity is adjusted to induce EPSCs with amplitude of 50–100 pA at 0.02 Hz and neurons were voltage clamped at -70 mV. The recording pipettes (3–5 MΩ) were filled with solution containing (mM): 145 K-gluconate, 5 NaCl, 1 MgCl_2_, 0.2 EGTA, 10 HEPES, 2 Mg-ATP, and 0.1 Na_3_-GTP (adjusted to pH 7.2 with KOH). Alexa fluor 594 (100 μM) was added in the intracellular solution to identify the expression of fosGFP as well as neuronal morphology. When current-voltage relationships were measured, K-gluconate was replaced by equomolar CsMeSO_3 _and 5 QX-314 chloride was added in the internal solution. Picrotoxin (100 μM) was always present to block GABA_A _receptor-mediated inhibitory currents and monitored throughout the synaptic currents. Access resistance was 15–30 MΩ and was monitored throughout the experiment. Data were discarded if access resistance changed more than 15% during an experiment. Statistical comparisons were performed using the Student's t-test or two way ANOVA test.

### Confocal imaging

To image GFP-positive neurons, a confocal microscope (Fluoview FV 1000, Olympus, Tokyo, Japan) was used [30]. The laser with a wavelength of 488 nm was used for GFP excitation and 633 nm was used for Alexa fluor 594.

### Immunohistochemistry

Ten animals, divided into two groups, were anaesthetized with isoflurane and perfused with 0.1 mol/L phosphate buffered saline (PBS, pH 7.2–7.4) via the ascending aorta followed by 4% paramaformaldehyde in 0.1 mol/L PB. The brains were then removed, and postfixed in the same fixative for 4 h before cryoprotection in PBS containing 30% sucrose overnight at 4°C. Every fourth sections of 25 μm thickness, serially cut through the brain in cryostat, were collected. Sections were then used for c-Fos and/or NeuN immunoreactivity.

Sections were sequentially incubated with the following solutions: (1) a solution of 3% bovine serum albumin (BSA, Sigma, St. Louis, USA), 0.3% Triton X-100 containing rabbit antibody against c-Fos (1:6000, Calbiochem, USA), or a mixture of rabbit antibody against c-Fos (1:6000, Calbiochem, USA) and mouse monoclonal antibody directed against NeuN (1: 400, Chemicon) for 2 days at 4°C, (2) Rhodamine-conjugated goat anti-rabbit (1:200, Chemicon), or a mixture of rhodamine-conjugated goat anti-rabbit (1:200, Chemicon) and FITC-conjugated goat anti-mouse (1:200, Jackson ImmunoResearch) antibodies in PBS containing 3% BSA and 0.3% Triton X-100 for 24 h at 4°C. For 3-3'-diaminobenzidine (DAB) reaction, biotinylated goat anti-rabbit IgG (1:200; Vector) was used in the second step and these sections were further incubated with avidin-biotin-complex (elite A, B; 1:200; Vector). All sections were rinsed with PBS (3 × 10 min) after each step. The signals were visualized under epifluorescence microscope or processed with DAB as chromogen using DAB kit (Vector, Laboratories, Burlingame, CA). No staining was observed on brain sections when the primary antibody was omitted from the protocol. Images were captured with the assistance of Image-Pro Plus 5.0 software, and all the parameters used were kept consistent during capturing. After microscopic observation, sections were then counterstained by Nissl technique for cytoarchitectural examination.

### Data analyses

Results were analyzed by t-test or two-way ANOVA followed by post-hoc Student-Newman-Keuls test to identify significant differences. All data are expressed as mean +/- SEM. In all cases, P < 0.05 was considered statistically significant.

## Competing interests

The authors declare that they have no competing interests.

## Authors' contributions

MZ designed the study. LJW, SSK, XL and FZ carried out experiments. LJW, SSK, XL and MZ wrote the manuscript.
